# Protective Effect of Tetrahydroxystilbene Glucoside on 6-OHDA-Induced Apoptosis in PC12 Cells through the ROS-NO Pathway

**DOI:** 10.1371/journal.pone.0026055

**Published:** 2011-10-06

**Authors:** Lizhen Tao, Xiaofeng Li, Lingling Zhang, Jiyu Tian, Xiaobing Li, Xin Sun, Xuefen Li, Lin Jiang, Xiaojun Zhang, Jianzong Chen

**Affiliations:** 1 Research Center of Traditional Chinese Medicine, Xijing Hospital, Fourth Military Medical University, Xi'an, People's Republic of China; 2 Department of Physics and Mathematics, School of Biomedical Engineering, Fourth Military Medical University, Xi'an, People's Republic of China; National Institutes of Health, United States of America

## Abstract

Oxidative stress plays an important role in the pathogenesis of neurodegenerative diseases, such as Parkinson's disease. The molecule, 2,3,5,4′-tetrahydr- oxystilbene-2-O-β-D-glucoside (TSG), is a potent antioxidant derived from the Chinese herb, Polygonum multiflorum Thunb. In this study, we investigated the protective effect of TSG against 6-hydroxydopamine-induced apoptosis in rat adrenal pheochromocytoma PC12 cells and the possible mechanisms. Our data demonstrated that TSG significantly reversed the 6-hydroxydopamine-induced decrease in cell viability, prevented 6-hydroxydopamine-induced changes in condensed nuclei and decreased the percentage of apoptotic cells in a dose-dependent manner. In addition, TSG slowed the accumulation of intracellular reactive oxygen species and nitric oxide, counteracted the overexpression of inducible nitric oxide syntheses as well as neuronal nitric oxide syntheses, and also reduced the level of protein-bound 3-nitrotyrosine. These results demonstrate that the protective effects of TSG on rat adrenal pheochromocytoma PC12 cells are mediated, at least in part, by the ROS-NO pathway. Our results indicate that TSG may be effective in providing protection against neurodegenerative diseases associated with oxidative stress.

## Introduction

Parkinson's disease (PD), the second most common neurodegenerative disorder after Alzheimer's disease (AD), is mainly characterized by progressive loss of dopaminergic (DAergic) neurons in the substantia nigra pars compacta (SNpc) [1], [2]. The cause of DAergic neuron loss in PD patients remains unexplained. However, several lines of evidence in PD patients and animal models have suggested that oxygen-free radicals and oxidative stress are involved in the pathogenesis of PD [3]–[5]. Thus, the regulation of oxidative stress may reduce or prevent the loss of DAergic neurons in PD patients. Medicinal herbs that have antioxidative effects are now being considered as therapeutic agents against neuronal loss [6]–[8].

Six-Hydroxydopamine (6-OHDA), a hydroxylated analog of the natural neurotransmitter, dopamine [9], can induce massive oxidative stress leading to the damage of DAergic neurons *in vitro* and *in vivo* [10]–[12]. PC12 cells, a cell line derived from rat adrenal pheochromocytoma cells, possess intracellular substrates for dopamine synthesis, metabolism and transport [10]. The apoptosis of PC12 cells induced by 6-OHDA has been used as an *in vitro* experimental model for the study of PD [13], [14].

**Figure 1:**
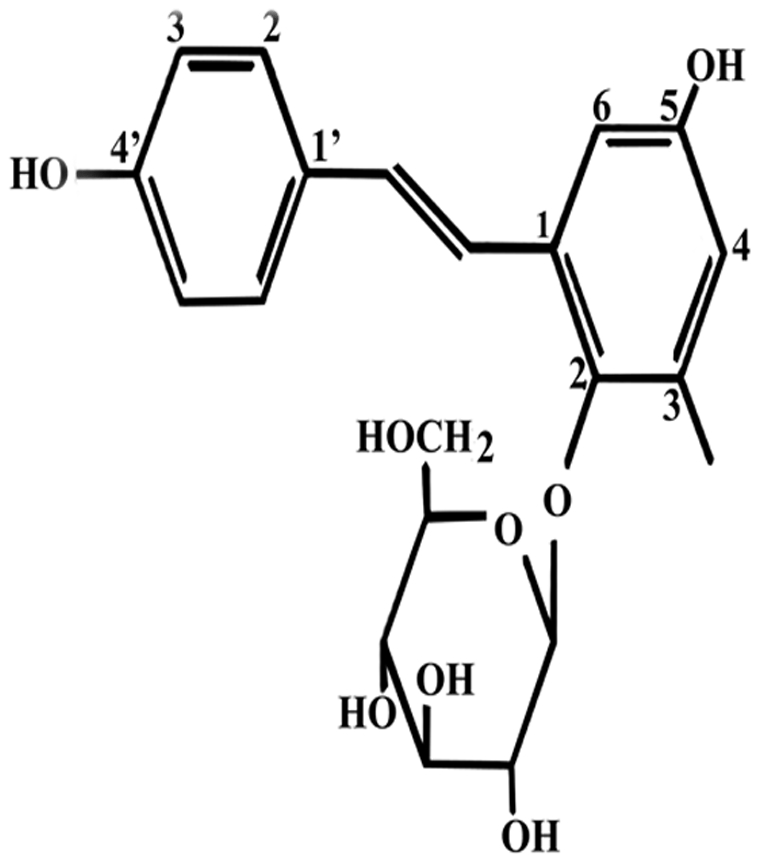
The chemical structure of TSG.

The root of *Polygonum multiflorum* Thunb (PM) is a widely used traditional Chinese herbal medicine. Some studies have suggested that PM and its extracts can be used to treat age-related diseases [15]–[17]. A monomer of stilbene, 2,3,5,4′-tetrahydroxy stilbene-2-O-*β*-D-glucoside (TSG) (Figure 1) is one of the main active ingredients of PM [18], [19]. TSG has been reported to have many pharmacological effects such as anti-oxidative and anti-inflammatory effects as well as improving memory and learning ability [20]–[22]. Luo et al. have shown that TSG may reduce the cognitive impairment and overexpression of amyloid precursor protein induced by exposure to aluminium and may have therapeutic effects against AD [23]. Moreover, TSG has been shown to possess neuroprotective effects against ischemia/reperfusion injury *in vitro* and *in vivo* [24]. Recently, our study group found that TSG may attenuate the 1-methyl-4-phenylpyridinium-induced apoptosis of PC12 cells by inhibiting reactive oxygen species (ROS) generation, and modulating the activation of Jun N-terminal kinase (JNK) and the PI3K/Akt pathway [25], [26].

In order to further investigate the neuroprotective effect of TSG, the present study aimed to evaluate the effects of TSG on PC12 cell viability and apoptosis induced by 6-OHDA. Additionally, we studied the possible mechanisms of TSG protection by measuring intracellular ROS, intracellular nitric oxide (NO), protein levels of inducible nitric oxide synthase (iNOS) and neuronal nitric oxide synthase (nNOS) and the level of 3-nitrotyrosine (3-NT).

## Materials and Methods

### 1. Materials

TSG (dissolved in distilled water, molecular weight 406, purity >98%) was obtained from the National Institute for the Control of Pharmaceutical and Biological Products (Beijing, China). 6-Hydroxydopamine hydrochloride (6-OHDA), 3-(4,5-dimethylthia- zol-2-yl)-2,5-diphenyltetra-zoliumbromide (MTT) and Hoechst 33258 were purchased from Sigma (St. Louis, MO, USA). Dulbecco's modified Eagle's medium (DMEM), fetal calf serum and horse serum were purchased from Gibco (Gaithersburg, MD, USA). Rabbit polyclonal antibodies to nNOS and to iNOS were purchased from Abcam Company (Cambridge, UK). A 3-NT ELISA kit was purchased from the Xitang Institute of Biotechnology (Shanghai, China). N^G^-methyl-L-arginine acetate salt (L-NMMA), glutathione (GSH), 3-amino-4-aminomethyl-2′,7′-difluorescein diacetate (DAF-FM DA) and the Reactive Oxygen Species Assay Kit were purchased from the Beyotime Institute of Biotechnology (Shanghai, China).

### 2. Methods

#### 2.1. Cell culture and treatment

PC12 cells are a cell line of rat adrenal pheochromocytoma cells that possess dopamine synthesis, metabolism and transporting system [26]. PC12 cells , kindly provided by People's Liberation Army(PLA) Institute of Neurobiology of the Fourth Military Medical University, were cultured in DMEM supplemented with 10% heat-inactivated horse serum, 5% heat-inactivated fetal calf serum, 100 IU/ml penicillin and 100 µg/ml streptomycin. Culture medium was changed every three days. PC12 cells were differentiated by 50 ng/mL nerve growth factor (NGF), which was added to the culture medium and incubated for a further 9 days, as described previously [27]. When cells reached 70% confluence, they were passaged in a ratio of 1∶3. In the experiments, cells were treated with different concentrations of 6-OHDA (25, 50, 75, 100, 125, 150, 200, 300 µM) for 24 h to investigate the neurotoxicity of 6-OHDA. TSG (10, 20, 50 µM) was added into cells for 24 h and then incubated with 75 µM 6-OHDA for 24 h. Control cells were treated in the same way without adding 6-OHDA and TSG to the culture medium.

#### 2.2. Exposure of PC12 cells to 6-OHDA with or without test substances

Cell viability was measured by MTT assay. PC12 cells were seeded in 96-well plates at a concentration of 1×10^4^ cells per well. After an overnight incubation, PC12 cells were incubated with 6-OHDA for 24 h, and then washed with phosphate buffer solution (PBS) three times. Following that, 20 µl MTT solution (5 mg/ml in PBS) was added to each well and the cells were incubated at 37°C for 4 h. The medium was then removed, and 150 µl of dimethyl sulfoxide was added to dissolve the blue crystals that formed in the cells. After being jolted by the shaker for 15 min, the absorbance of the solution at 490 nm was measured in a microplate reader (Bio-Rad, USA). Control cells were treated in the same way without 6-OHDA treatment, and the values of different absorbances were expressed as a percentage of the control.

#### 2.3. Staining of nuclear DNA in apoptotic cells with Hoechst 33258

The changes in nuclear morphology of apoptotic cells were examined by Hoechst 33258 staining and visualized under a fluorescence microscope. After treatment with 6-OHDA and/or TSG for 24 h, PC12 cells were fixed with 4% paraformaldehyde for 30 min. Subsequently, PC12 cells were washed three times with PBS, and incubated with 3 µg/mL Hoechst 33258 for 30 min at room temperature in the dark. The cells were observed under a fluorescence microscope (Olympus IX71, Japan) after being washed twice with PBS. Cells that exhibited reduced nuclear size, chromatin condensation, intense fluorescence, and nuclear fragmentation were considered apoptotic.

#### 2.4. Flow cytometric analysis of cell apoptosis

In order to detect early apoptosis and late apoptosis/necrosis induced by 6-OHDA quantitatively, cell apoptosis was measured using the Annexin V-FITC Apoptosis Detection Kit (BD Pharmingen, USA) according to the manual. Briefly, after treatment with 6-OHDA and/or TSG for 24 h, PC12 cells were collected, washed, re-suspended in 100 µl binding buffer, and stained with 5 µl annexin V-FITC and 5 µl PI staining solution in the dark at room temperature for 15 min. The cell samples were analyzed by flow cytometry on a FACScan station with Cell Quest software using the FL1 and FL2 range for annexin V-FITC and PI, respectively.

#### 2.5. Measurement of intracellular ROS

ROS were measured with the 2′,7′-dichlorofluorescein diacetate (DCFH-DA) as previously described [28]. Briefly, after PC12 cells were treated with 6-OHDA and/or TSG for 24 h, the cells were washed three times with PBS. DCFH-DA, diluted to a final concentration of 10 µM, was added to PC12 cells and these were incubated for 30 min at 37°C in the dark. After the cells were washed three times with serum-free medium, the fluorescence intensity was detected with a multi-detection microplate reader with excitation at 488 nm and emission at 530 nm within 15 min. Control cells were treated in the same way without adding 6-OHDA and TSG to the culture medium. The measured fluorescence values were expressed as a percentage of the fluorescence in control cells.

#### 2.6. Measurement of intracellular NO

Intracellular NO was measured with 3-amino,4-aminomethyl-2′,7′-difluorescein, diacetate (DAF-FM DA), as previously described [29]. Briefly, after PC12 cells were treated with 6-OHDA and/or TSG for 24 h, the cells were washed three times with PBS and then incubated in 5 µM DAF-FM DA at 37°C for 20 min. After a further three PBS washes, the fluorescence intensity was analyzed by a multi-detection microplate reader with excitation at 495 nm and emission at 515 nm within 15 min. The measured fluorescence values were expressed as a percentage of the fluorescence in control cells.

#### 2.7. Measurement of protein-bound 3-NT

Protein-bound 3-NT was detected with the ELISA method according to the manual. Briefly, a nitrated protein solution was prepared and diluted for use as a standard. These standard samples and cell samples were pipetted into 96-well plates and incubated with a rabbit polyclonal anti-nitrotyrosine primary antibody at 37°C for 1 h. Following this, samples were incubated with a horseradish peroxidase-conjugated secondary antibody for 1 h and washed. Subsequently, these samples were incubated with freshly prepared LumiGLO Chemiluminescent Substrate for 10 min. Luminescence was then measured with a microplate reader (Bio-Rad, USA). The nitrotyrosine content in cell samples was calculated by standard curves generated from nitrated bovine serum albumin containing quantified nitrotyrosine amounts.

#### 2.8. Western blot analysis of nNOS and iNOS

After PC12 cells were treated with 6-OHDA and/or TSG, cells were solubilized with 5×SDS (sodium dodecyl sulfate) loading buffer (0.25 mM Tris-HCl, pH 6.8, containing 5% β-mercaptoethanol, 10% SDS, 50% glycerol, and 0.5% bromophenol blue) and boiled for 5 min in 100°C water. Twenty microliters of each sample were loaded into a lane of a 12% SDS-PAGE, and then electrophoretically transferred to polyvinylidene fluoride (PVDF) membranes. The membranes were blocked in blocking buffer (tris-buffered saline tween TBST containing 5% non-fat milk) for 2 h and incubated overnight with primary antibodies at 4°C. After triplicate washes with PBS/Tween 20, the membranes were incubated with secondary antibodies at 37°C for 2 h. Finally, the membranes were developed with enhanced chemiluminescence (ECL) substrate and exposed to X-ray film. Densitometry was performed using WorkLab software (UVP, USA). The data were recorded as the ratio of sample to β-actin.

### 3. Statistical analysis

All experiments were performed at least three times. Data are expressed as mean ± standard errors of mean (S.E.M.). Differences were analyzed with one-way analysis of variance (ANOVA) with subsequent post hoc analysis using Bonferroni differences where the data were considered statistically significant (*P*<0.05).

## Results

### 1. Effects of TSG on 6-OHDA-induced viability of PC12 cells

**Figure 2:**
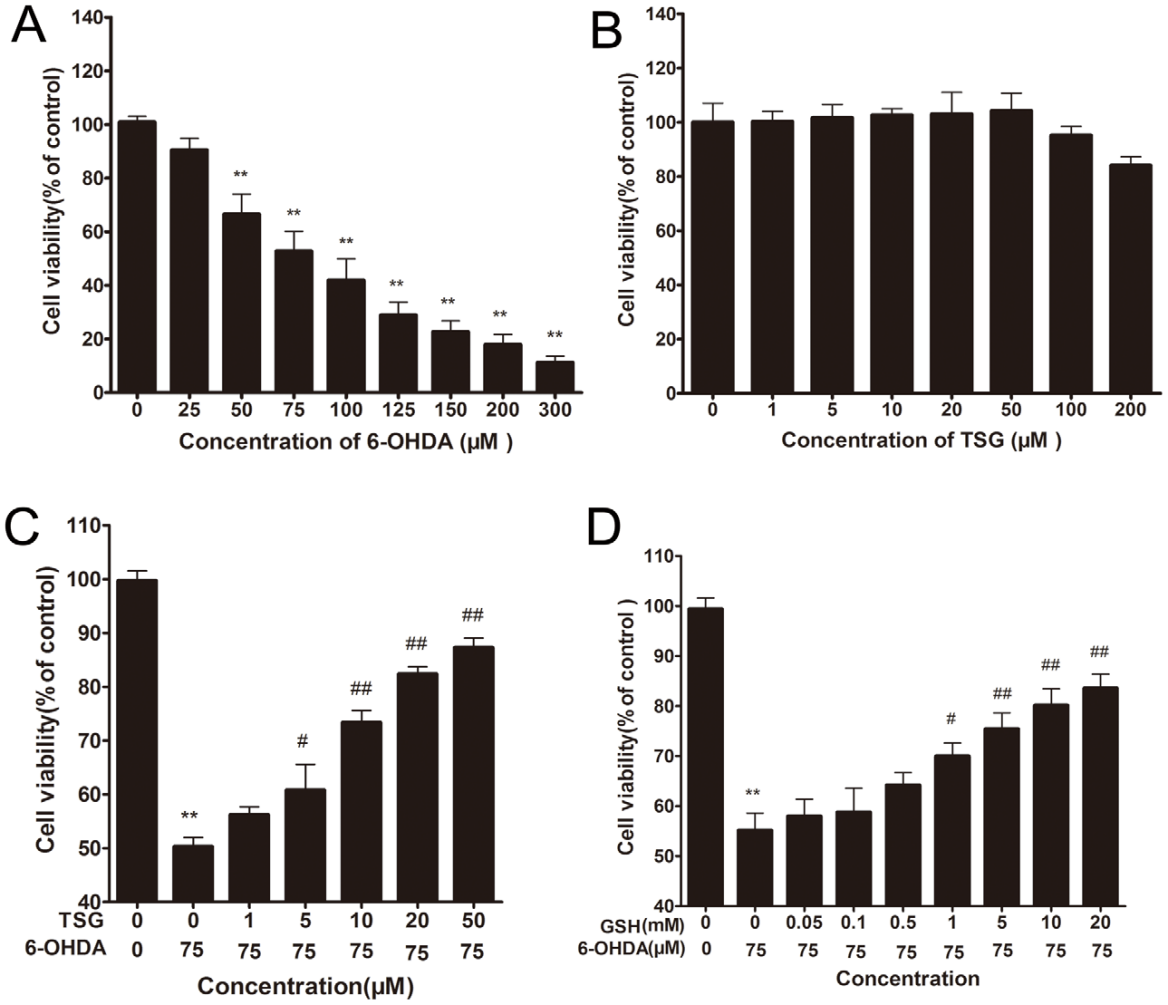
Effects of TSG and 6-OHDA on cell viability. Cells were incubated for 24 h in different concentrations of 6-OHDA alone (A) or in different concentrations of TSG alone (B). Cells were preincubated with different concentrations of TSG (C) for 24 h or GSH (D) for 1 h, after which 6-OHDA (75 µM) was added for 24 h. The data are expressed as percentage of untreated control cells ± standard deviation (n = 6). **P<0.01 versus untreated control cells; # *P*<0.05, # #*P*<0.01 versus 6-OHDA-treated cells.

There was a dose-dependent decrease in cell viability following 6-OHDA exposure to PC12 cells (Figure 2A). Cell viability significantly decreased by approximately 50% after PC12 cells were treated with 75 µM 6-OHDA for 24 h. Cells treated with various concentrations of TSG alone for 24 h showed no obvious effect on cell viability (Figure 2B). Conversely, cells treated with various concentrations of TSG for 24 h before the addition of 6-OHDA (75 µM) for 24 h showed that cell viability increased at concentrations of 10, 20, and 50 µM TSG (Figure 2C). This suggested that TSG has a positive effect on cell viability in a dose-dependent manner. As shown in Figure 2D, GSH, a well-known antioxidant molecule [30], could protect PC12 cells from 6-OHDA-induced loss of cell viability in a dose-dependent manner.

### 2. Effects of TSG on 6-OHDA-induced changes in nuclear morphology

**Figure 3:**
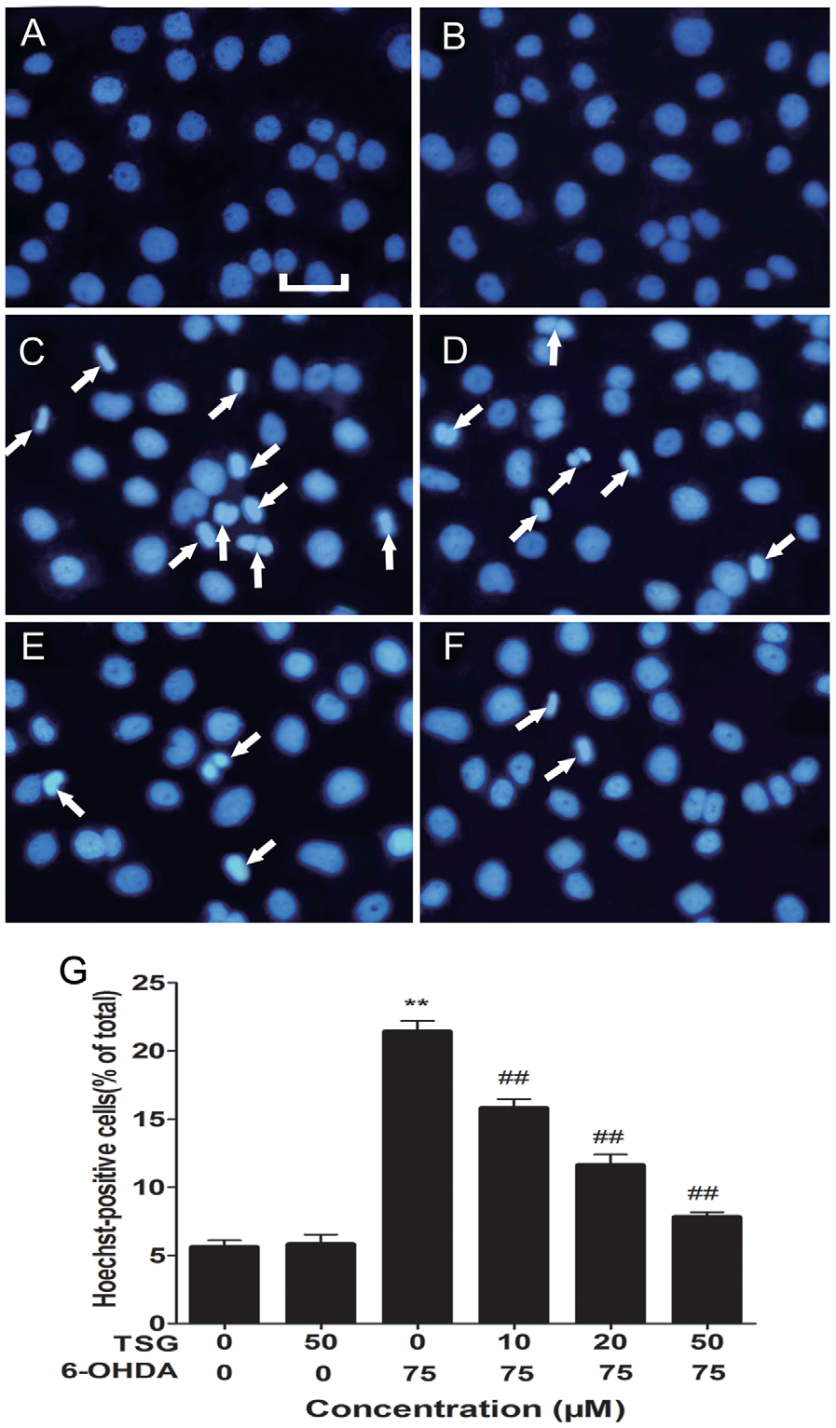
Fluorescence images show the nucleic changes of PC12 cells incubated in 6-OHDA with or without TSG. Cells were stained with the DNA-binding fluorochrome Hoechst 33258. (A) shows normal culture medium nucleic morphology, (B) and (C) respectively show cells cultured in 50 µM TSG or 75 µM 6-OHDA for 24 h. In addition, cells were pretreated with 10 µM (D), 20 µM (E) or 50 µM (F) TSG for 24 h and then incubated in 6-OHDA (75 µM) for an additional 24 h. (G) Histograms showing ratio of condensed nuclei to total nuclei. White arrows represent location of apoptosis cell. Scale bars represent 50 µm. **P<0.01 versus untreated control cells; # #*P*<0.01 versus 6-OHDA-treated cells.

Changes in nuclear morphology were tested by Hoechst 33258 staining. The normal nucleus showed a homogeneous staining, bearing regular contors and rounded shapes (Figure 3A and B). Most cells showed an asymmetrical, highly bright fluorescence, and the number of condensed nuclei increased after exposed to 75 µM 6-OHDA for 24 h (Figure 3C). These changes in the nuclear morphological character were prevented significantly by pretreatment of cells with TSG (Figure 3D–F), especially at concentrations of 20 µM (Figure 3E) and 50 µM (Figure 3F) TSG. However, TSG alone had no effect on nuclear morphology (Figure 3B).

### 3. Effects of TSG on 6-OHDA-induced percentage of apoptosis

**Figure 4:**
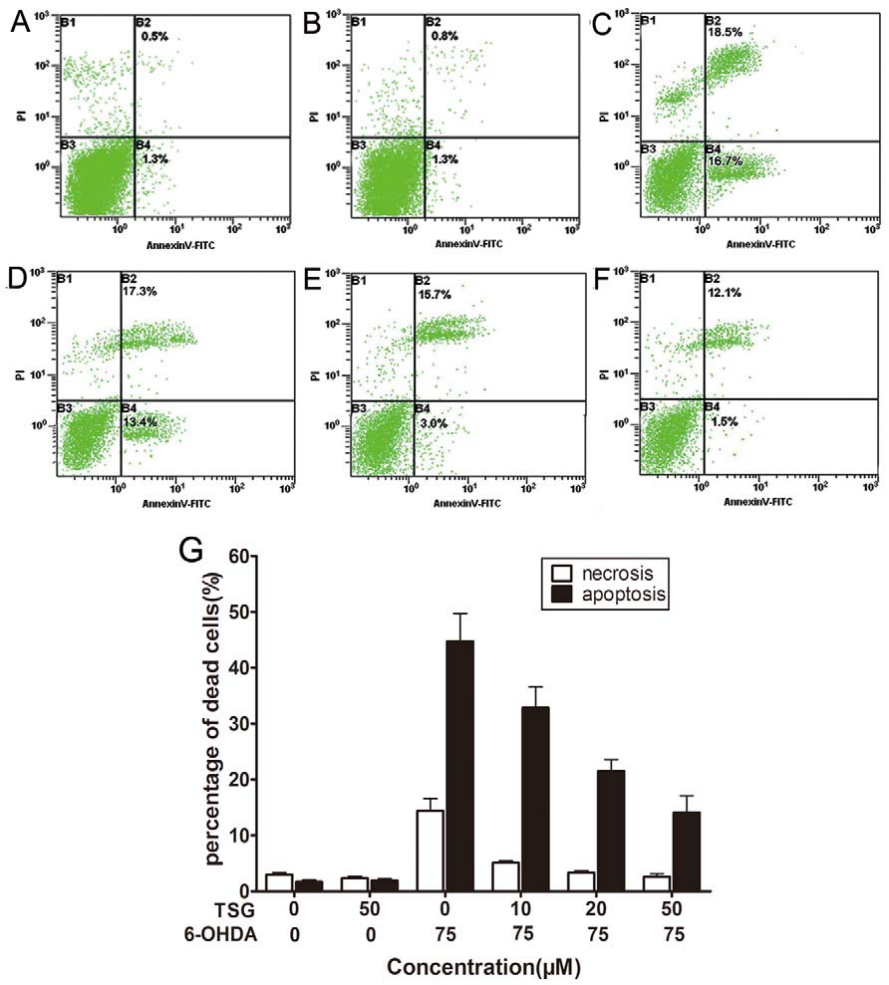
Cell apoptosis and necrosis detected by flow cytometry. PC12 cells were incubated in drug-free medium (A) or medium containing 50 µM TSG (B) or 75 µM 6-OHDA (C) for 24 h; or cells were pretreated with 10 µM (D), 20 µM (E) or 50 µM (F) TSG for 24 h and then incubated in 6-OHDA (75 µM) for an additional 24 h. The results shown in (G) are the mean and SE for three independent experiments.

The annexin-V^-^/PI^-^ population is made up of normal healthy cells, while annexin-V^+^/PI^-^ cells exist in early apoptotic stage, and annexin-V^+^/PI^+^ cells exist in late apoptotic/necrotic stage. After 24 h of incubation with 75 µM 6-OHDA, our results showed that the percentage of early apoptosis increased significantly (Figure 4C), and TSG showed a positive effect on apoptosis in a dose-dependent manner (Figure 4D–F). TSG alone did not display any obvious effect (Figure 4B).

### 4. Effects of TSG on intracellular ROS levels

**Figure 5:**
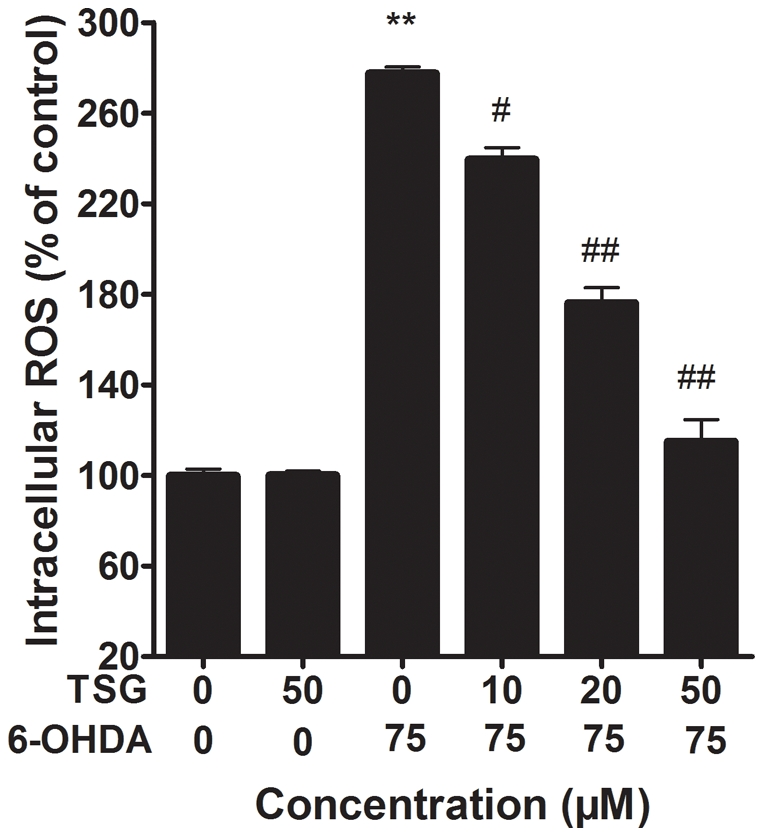
Effect of TSG on 6-OHDA-induced accumulation of intracellular ROS levels. Cells were pretreated with different concentrations of TSG for 24 h and then incubated in 6-OHDA for an additional 24 h. Intracellular ROS levels were measured using DCFH-DA. Data are expressed as percentage of untreated control cells ± standard deviation (n = 6). **P<0.01 versus untreated control cells; # #*P*<0.01 versus 6-OHDA-treated cells.

As shown in Figure 5, exposure of PC12 cells to 75 µM 6-OHDA for 24 h led to a 2.7-fold accumulation in the level of intracellular ROS, compared with the control group (*P*<0.01). Such accumulation in the level of intracellular ROS was significantly reduced in a dose-dependent manner by pretreatment of the cell with TSG. Additionally, TSG alone had no obvious effect on intracellular ROS levels.

### 5. Effects of TSG on the levels of intracellular NO

**Figure 6:**
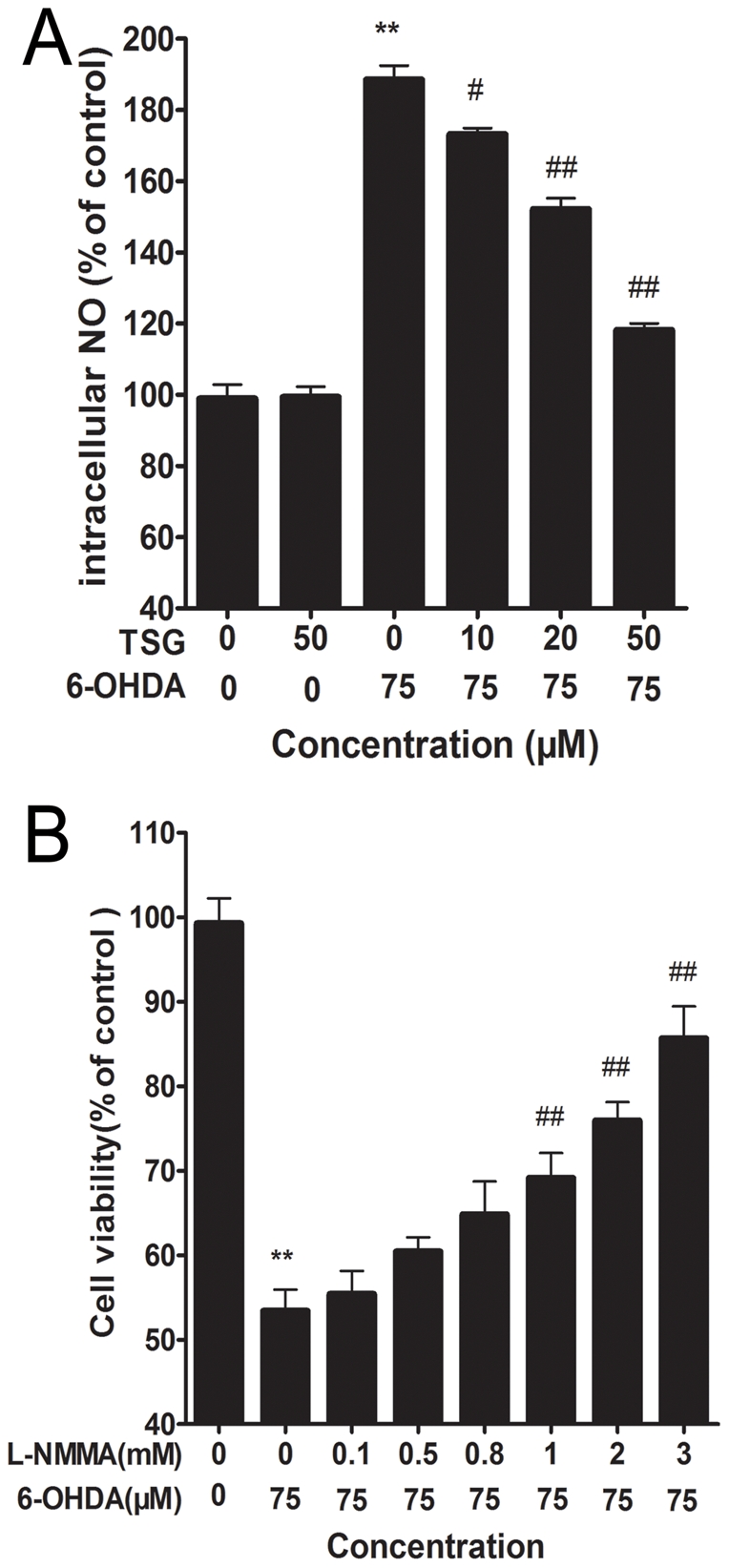
Effect of TSG on 6-OHDA-induced accumulation of intracellular NO (A). Cells were exposed to 6-OHDA with or without different concentrations of TSG for 24 h. (B) Effect of L-NMMA on 6-OHDA-induced cell viability in PC12 cells. Cells were pretreated with different concentrations of L-NMMA for one hour and then incubated in 6-OHDA for an additional 24 h. Data are expressed as percentage of untreated control cells ± standard deviation (n = 6). **P<0.01 versus untreated control cells; # #*P*<0.01 versus 6-OHDA-treated cells.

Exposure of PC12 cells to 75 µM 6-OHDA for 24 h led to a rapid increase in DAF-FM fluorescence, compared with the control group (*P*<0.01) (Figure 6A). TSG (20 and 50 µM) pre-treatment inhibited such increase in DAF-FM fluorescence, while TSG alone had no effect on DAF-FM fluorescence intensity (Figure 6A). As shown in Figure 6B, L-NMMA, a NOS inhibitor, protected PC12 cells from 6-OHDA-induced loss of cell viability in a dose-dependent manner. These results may also indicate that the protective effect of TSG on 6-OHDA-induced PC12 cell viability is through prevention of elevation in intracellular NO levels.

### 6. Effects of TSG on the level of protein-bound 3-NT

**Figure 7:**
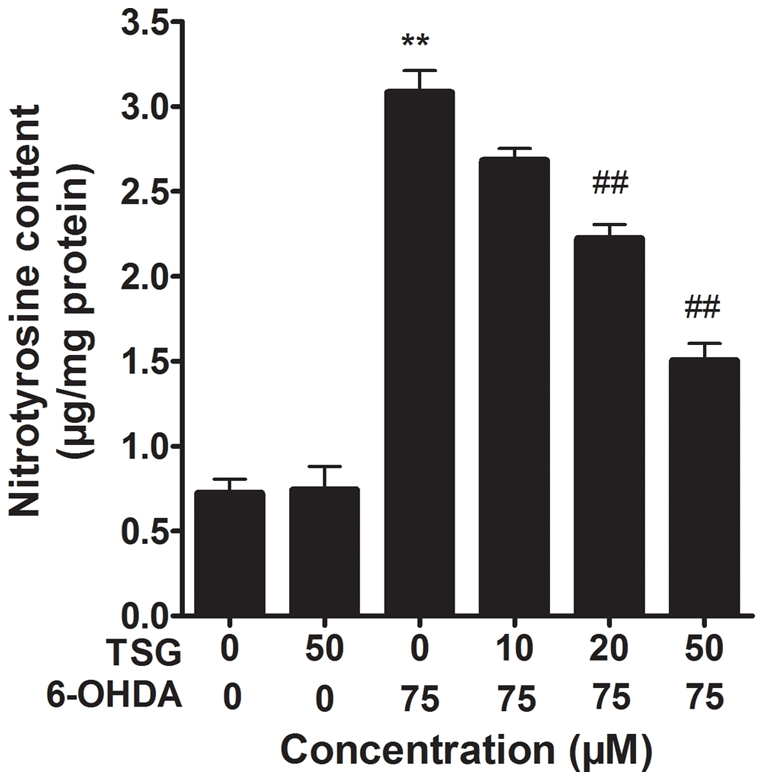
Effect of TSG on 6-OHDA-induced elevation of protein-bound 3-NT in PC12 cells. PC12 cells were exposed to TSG (10, 20 or 50 µM) for 24 h before 75 µM 6-OHDA was added to the medium for an additional 24 h, and then 3-NT was measured. Data are expressed as percentage of untreated control cells ± standard deviation (n = 5). **P<0.01 versus untreated control cells; # *P*<0.05, # #*P*<0.01 versus 6-OHDA-treated cells.

As shown in Figure 7, protein-bound 3-NT was measured by a competitive ELISA method with an anti-3-NT antibody. After 6-OHDA treatment, the level of 3-NT increased approximately 4.5-fold compared with the control group (*P*<0.01). TSG pretreated cells showed a reverse effect in a dose-dependent manner in the 10, 20 and 50 µM ranges, compared with the 6-OHDA group. TSG alone had no obvious effect on the protein-bound 3-NT levels in PC12 cells.

### 7. Effects of TSG on the expression of nNOS and iNOS

**Figure 8:**
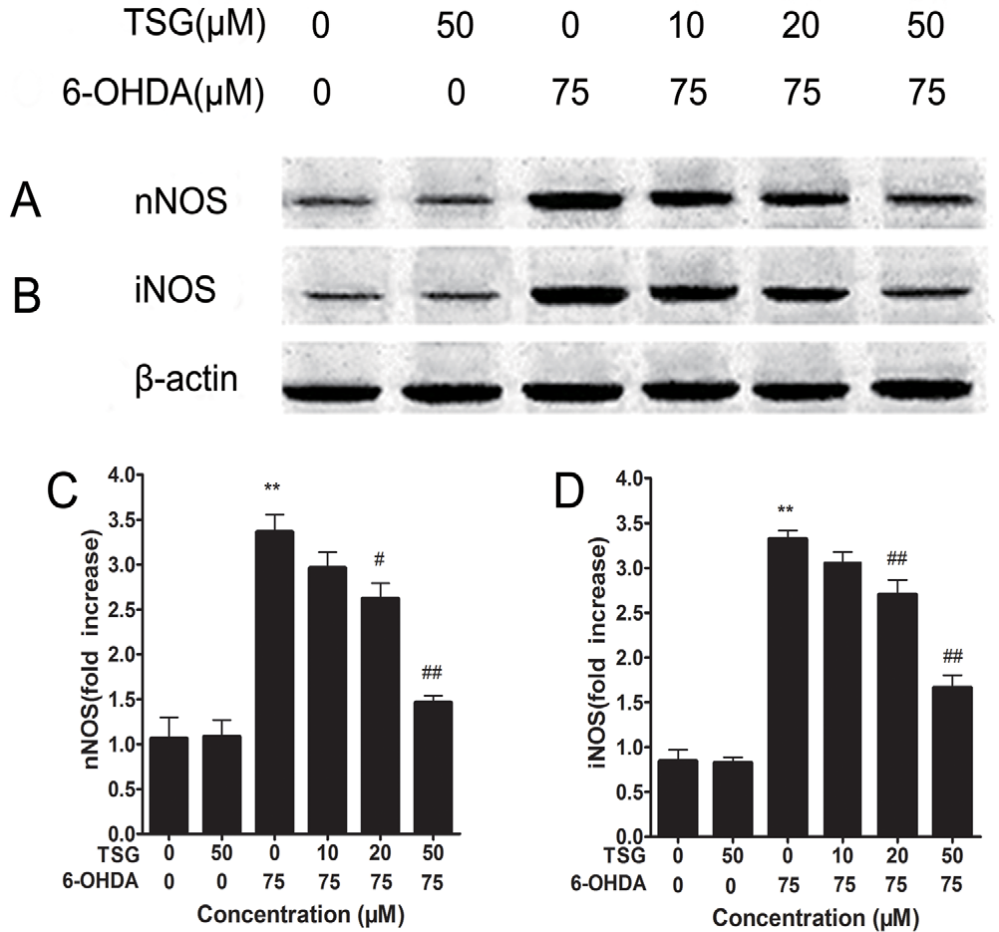
Effect of TSG and 6-OHDA on the expression of nNOS and iNOS. PC12 cells were exposed to 75 µM 6-OHDA with or without different concentrations of TSG for 24 h, and the nNOS (A) and the iNOS (B) were detected by Western blotting. (C) and (D) show the quantitative analysis of nNOS and iNOS protein levels, respectively. Data obtained from quantitative densitometry were presented as mean ± standard deviation of three independent experiments. *P<0.05, **P<0.01 versus untreated control cells; # *P*<0.05, # #*P*<0.01 versus 6-OHDA treated cells.

Western blotting reflected that 6-OHDA induced a 3.2-fold increase in the immunoreactivity of nNOS in PC12 cells compared with control cells. Pre-treatment with TSG reduced the expression of nNOS induced by 6-OHDA in a dose-dependent manner (Figure 8A and C). Additionally, 6-OHDA induced a 3.5-fold increase in the immunoreactivity of iNOS in PC12 cells. Pretreatment with TSG reduced the expression of iNOS induced by 6-OHDA in a dose-dependent manner (Figure 8B and D). Treatment with 50 µM TSG alone had no obvious influence on the expression of iNOS and nNOS in PC12 cells.

## Discussion

Our study showed that exposure of PC12 cells to 75 µM 6-OHDA for 24 h significantly reduced cell viability, induced typical apoptosis features such as nuclei concentration, boosted the percentage of apoptosis cells, increased the level of intracellular ROS and NO, induced overexpression of iNOS and nNOS and elevated the level of 3-NT. However, the above changes were markedly reversed in a dose-dependent manner after the pre-treatment of PC12 cells with different concentrations of TSG for 24 h, which suggests that TSG may protect PC12 cells from 6-OHDA-induced apoptosis by regulation of ROS-NO pathway.

It is well known that the pathogenesis of age-related diseases such as PD involves the generation of ROS [31]. The mechanism of 6-OHDA toxicity is linked to production of extracellular ROS via auto-oxidation and generation of intracellular ROS after being taken up by PC12 cells [32]–[34]. The generation of intracellular ROS by 6-OHDA is either by direct inhibition of mitochondrial respiratory chain complexes I and IV or by enzymatic deamination through monoamine oxidase [35]. Moreover, the generation of intracellular ROS by 6-OHDA is an initial event and the ROS suppresses the Akt phosphorylation, increases p38 phosphorylation which induces the activation of caspase-9 as well as caspase-3, and finally leads to cell apoptosis [32]. These indicate that ROS plays an important role in cell apoptosis induced by 6-OHDA [8]. Our results showed that the level of intracellular ROS significantly increased after PC12 cells were treated with 6-OHDA for 24 h. However, the level of intracellular ROS decreased in a dose-dependent manner when PC12 cells were pretreated with different concentrations of TSG prior to 6-OHDA treatment.

NO is synthesized from L-arginine by NOS in the presence of reduced nicotinamide adenine dinucleotide phosphate and molecular oxygen [36]. NO is a well known vasorelaxant agent, while NO produced by neurons plays an important role as a neurotransmitter and NO produced by immune and glial cells is involved in defense functions [37]. In addition, in the brain, metabolism of NO seems to be indispensable for normal cerebral function such as pain perception, synaptic plasticity and learning. However, NO may act as a neurotoxin and cause neuronal injury and death when inappropriate/excessive NO is produced in the brain [36], [38]–[40]. Moreover, there is increasing evidence that NO is involved in the pathogenesis of neurodegenerative diseases, including PD [41]–[43]. To date, NOS has four known isoforms: nNOS, iNOS, eNOS, and mitochondrial nitric oxide synthase [37], [44]. nNOS and iNOS are acknowledged to be closely related to the pathogenesis of PD, for example, nNOS inhibitor has a dose-dependent protective effect against 1-methyl-4- phenyl-1,2,3,6- tetrahydropyridine (MPTP)-induced striatal dopamine and 3,4- dihydroxyphenylacetic acid depletion in mice, and dopaminergic neurons in the SNPc of iNOS-deficient mice were almost completely protected from MPTP toxicity in a chronic paradigm of MPTP toxicity [45], [46]. Our Western blot analyses showed that the expression of nNOS and iNOS proteins increased after treatment of PC12 cells with 6-OHDA for 24 h. We also found the level of intracellular NO to rise significantly after PC12 cells were treated with 6-OHDA for 24 h. Conversely, both the level of intracellular NO and the expression of nNOS as well as iNOS proteins decreased in a dose-dependent manner after pre-treatment of PC12 cells with different concentrations of TSG. Additionally, inhibiting the generation of NO by L-NMMA can partly counteract the decrease in cell viability induced by 6-OHDA. These data suggest that NO plays an important role in cell apoptosis induced by 6-OHDA. TSG, like L-NMMA, may inhibit the generation of NO and decrease the level of cell apoptosis induced by 6-OHDA.

NO can cause neuronal cell death though different pathways including activation of p53, increase of endoplasmic reticulum stress, activation of mitochondrial permeability transition and subsequent release of cytochrome c, and activation of p38 or other MAP kinase pathways [47]. Notably, excessive NO can react with ROS rapidly enough to avoid the action of antioxidant systems, forming peroxynitrite anion (ONOO-), which can inhibit or damage the mitochondrial complexes I, II, IV and V, mitochondrial membrane, mitochondrial DNA and superoxide dismutase and induce mitochondrial swelling and Ca2+ release, which stimulates Ca2+-dependent enzymes and triggers apoptosis [37], [48]–[52]. Moreover, Guo et al. found that 6-OHDA elevated the level of 3-NT in SH-SY5Y cells [6]. It is hard to detect ONOO-, thus, the generation of ONOO- is usually measured by levels of 3-NT, a footprint of ONOO- [53]. We found that 6-OHDA elevated the level of 3-NT in PC12 cells, while the level of 3-NT decreased after pretreatment of PC12 cells with TSG.

In summary, our data indicate that TSG protects PC12 cells from 6-OHDA-induced apoptosis. The protective effects of TSG are mediated, at least in part, by regulating the ROS-NO pathway. Our results suggest that TSG could be used as a neuroprotective agent in the treatment of neurodegenerative diseases.
